# Evaluating overall survival and competing risks of survival in patients with early‐stage breast cancer using a comprehensive nomogram

**DOI:** 10.1002/cam4.3030

**Published:** 2020-04-20

**Authors:** Yan‐Bo Xu, Hong Liu, Qi‐Hua Cao, Jia‐Li Ji, Rong‐Rong Dong, Dong Xu

**Affiliations:** ^1^ Department of Surgical Oncology The Second Affiliated Hospital of Zhejiang University School of Medicine Hangzhou China; ^2^ Department of Medical Oncology The Second Affiliated Hospital of Zhejiang University School of Medicine Hangzhou China; ^3^ Department of Oncology Affiliated Cancer Hospital of Nantong University Nantong China; ^4^ Department of Medical The Children’s Hospital of Zhejiang University School of Medicine Hangzhou China; ^5^ Department of Surgical Oncology and Cancer Institute The Second Affiliated Hospital of Zhejiang University School of Medicine Hangzhou China

**Keywords:** breast cancer, cancer‐specific survival, nomogram, overall survival

## Abstract

**Background:**

Patients with early‐stage breast cancer (BC) live long but have competing comorbidities. This study aimed to estimate the effect of cancer and other causes of death in patients with early‐stage BC and further quantify the survival differences.

**Materials and methods:**

Data of patients diagnosed with BC between 2010 and 2016 were collected from the Surveillance, Epidemiology, and End Results database. The cumulative incidence function for breast cancer–specific mortality (BCSM) and other cause‐specific mortality (OCSM) was estimated, and the differences were tested using the Gray test. The nomogram for estimating 3‐, 4‐, and 5‐year overall survival (OS), breast cancer–specific survival, and other cause‐specific survival was established based on Cox regression analysis and Fine and Gray competing risk analysis. The discriminative ability, calibration, and precision of the nomogram were evaluated and compared using C statistics, calibration plots, and area under the receiver operating characteristic curve.

**Results:**

A total of 196 304 eligible patients with early‐stage BC were identified in this study. Of these, 12 417 (6.3%) patients died: 5628 (45.3%) due to BC and 6789 (54.7%) due to other causes. Five validated variables were incorporated to develop the prognostic nomogram: age, grade, tumor size, subtype, and surgery of primary site (Figure 3). Age was a strong predictive factor, which was more obvious in OCSM. The effect of surgery was more prominent in BCSM. Increased tumor size was correlated with OS and BCSM and slightly correlated with OCSM. Grade and subtype differences were more predominant in BCSM than in OCSM. The established nomogram was well calibrated and displayed good discrimination.

**Conclusions:**

We evaluate OS and competing risks of death in patients with early‐stage BC, establishing the first comprehensive prognostic nomogram.

## Graphical abstract

Our study analyzed the survival and mortality of early‐stage BC, discriminated the difference between cancer and non‐cancer‐related risk factors. This is the first study using Fine and Gray’s competing risk analysis and build a comprehensive nomogram. The established nomogram was well calibrated, and displayed good discrimination.

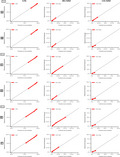

## 1 INTRODUCTION

Breast cancer (BC) is the most common malignant tumor in women and the main cause of cancer‐specific death, with 268 600 estimated new cases and 41 700 estimated deaths in 2019 in the USA.^1^ Presently, the prognosis of BC, especially early‐stage BC, has been dramatically improved by multidisciplinary treatments, including radical resection, neo‐/adjuvant chemotherapy, and hormone and targeted therapies.^2^, ^3^, ^4^ In developed countries, early‐stage BC has become the most frequently diagnosed invasive breast disease. However, in BC survivors, comorbidities, such as cardio‐ and cerebrovascular diseases, compete with BC as primary causes of death. Given the good prognosis of early‐stage BC, the long‐term benefit of treatment, particularly in the elderly population, depends on competing risks of death. Thus, considering the competing risks is necessary in the assessment of prognosis. Several published studies ^5^, ^6^, ^7^, ^8^, ^9^ have reported the prognosis of BC, but most of them only either paid more attention on overall survival (OS) or analyzed cancer‐specific mortality using the traditional Cox regression model, which cannot necessarily reflect the effect on cumulative incidence. In the individualized treatment era, evaluating the OS is not far enough. It is important to differentiate cancer‐specific and other cause‐specific mortality (OCSM). When competing risks exist, the traditional Cox regression model may be inappropriate because, in this model, the competing events are regarded as censoring and cancer‐specific mortalities may be overestimated.^10^, ^11^, ^12^ Thus, in this situation, the Fine and Gray model^11^, ^13^ is recommended.

Therefore, we evaluate OS and competing risks of death (BC related and other causes related) in patients with early‐stage BC and build a comprehensive nomogram to provide the physician with a quantitative tool using a large population of early‐stage BC.

## 2 MATERIALS AND METHODS

### 2.1 Patients

**FIGURE 1:**
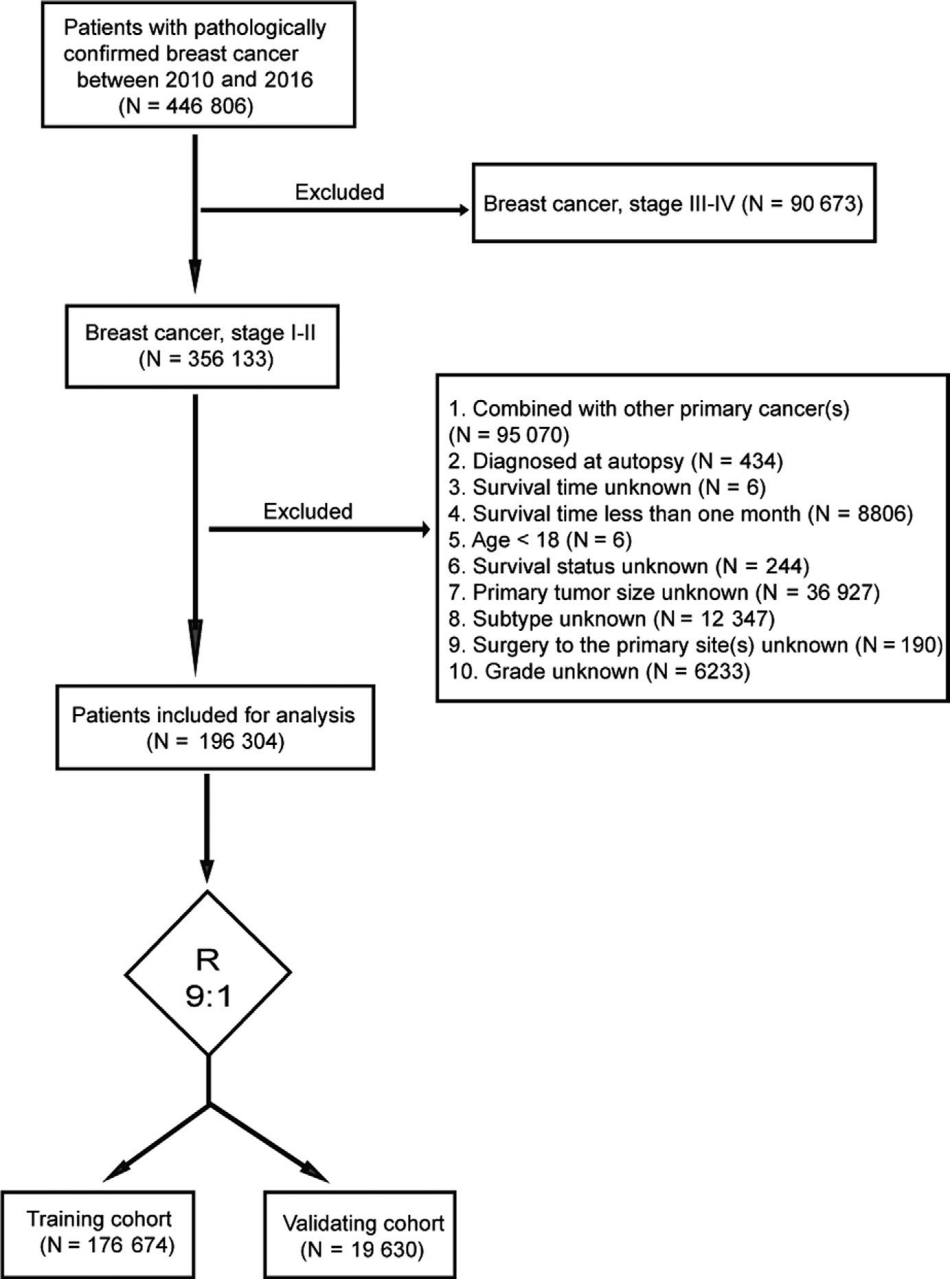
Flowchart of patient selection

Data of patients with early‐stage (stage I‐II) BC were retrospectively extracted from the Surveillance, Epidemiology, and End Results (SEER) database (2010‐2016) using SEER*Stat version 8.3.4. We identified a total of 446 806 patients who were pathologically diagnosed with BC. The exclusion criteria were as follows: (a) stage III‐IV BC (N = 90 673); (b) other primary cancers (N = 95 070); (c) diagnosis at autopsy (N = 434); (d) censored or incomplete information on survival time (N = 6), survival status (N = 244), primary tumor size (N = 36 927), subtype (N = 12 347), primary site surgery (N = 190), and grade (N = 6233); (e) survival time <1 month (N = 8806); and (f) age <18 years (N = 6). Thus, 196 304 patients were included in the final cohort for analysis. The detailed patient selection criteria are shown in Figure 1. Informed consent was not required because the SEER database does not contain personal information. Clinicopathological variable selection depended on clinical importance and predictors identified in previous studies,^6^, ^8^, ^9^ including age, grade, tumor size, subtype, surgery to primary sites, and survival time. We classified age at diagnosis into seven groups: <60, 60‐65, 66‐70, 71‐75, 76‐80, 81‐85, and >85 years. The tumor sizes were categorized into five groups (<1, <2, <3, <4, and ≥4 cm) for the OS analysis, six groups (<1, <2, <3, <4, <5, and ≥5 cm) for the breast cancer‐specific mortality (BCSM) analysis, and four groups (<1, <2, <3, and ≥3 cm) for the OCSM analysis. Subsequently, the 196 304 patients with stage I–II BC were randomly divided into two groups at a ratio of 9:1, training cohort (N = 176 674) and validation cohort (N = 19 630), using random number method produced by runif function of stats R package. The training cohort was used to construct the nomogram, while the validation cohort was used for validation. There were no significant differences between the two groups (*P* > .05) (Table 1).

**TABLE 1:** Patient characteristics of the training cohort and the validation cohort

	Training cohort No. (%)	Validation cohort No. (%)	*P*‐value
Age (y)			0.702
<60	83 835 (42.7)	9312 (4.7)	
60‐65	30 234 (15.4)	3394 (1.7)	
66‐70	22 145 (11.3)	2493 (1.3)	
71‐75	16 528 (8.4)	1813 (0.9)	
76‐80	11 461 (5.8)	1290 (0.7)	
81‐85	7374 (3.8)	772 (0.4)	
>85	5097 (2.6)	556 (0.3)	
Grade			0.996
I	44 718 (22.8)	4973 (2.5)	
II	78 434 (40.0)	8716 (4.4)	
III‐IV	53 522 (27.3)	5941 (3.0)	
Tumor size (cm)			0.841
<1	41 174 (21.0)	4598 (2.3)	
1‐1.9	69 697 (35.5)	7687 (3.9)	
2‐2.9	37 264 (19.0)	4169 (2.1)	
3‐3.9	16 006 (8.2)	1804 (0.9)	
4‐4.9	7480 (3.8)	836 (0.4)	
≥5	5053 (2.6)	536 (0.3)	
Subtype			0.453
HR−/HER2− (triple negative)	19 017 (9.7)	2183 (1.1)	
HR−/HER2+ (HER2 enriched)	6976 (3.6)	760 (0.4)	
HR+/HER2− (luminal A)	132 675 (67.6)	14 708 (7.5)	
HR+/HER2+ (luminal B)	18 006 (9.2)	1979 (1.0)	
Surgery to primary sites			0.901
No	5268 (2.7)	589 (0.3)	
Yes	171 406 (87.3)	19 041 (9.7)	

Abbreviations: HER2, human epidermal growth factor receptor type 2; HR, hormone receptor; No., number.

### 2.2 Statistical analysis

Demographic and clinical characteristics were summarized using descriptive statistics. Categorical variables were reported as whole numbers and proportions, and continuous variables were reported as medians with interquartile ranges (IQRs), unless indicated otherwise. The chi‐square test and Fisher's exact test for categorical variables and Student's *t* test for continuous variables were performed to compare baseline characteristics.

OS was defined as the time from diagnosis to death by any cause. The Kaplan‐Meier method was used to generate OS, and the log‐rank test was used to examine the differences in OS. The associations between relevant clinical variables and OS were analyzed using the Cox regression model.

We used the cumulative incidence function (CIF) to describe cause‐specific survival and Gray's test to analyze the differences. We classified cause of death as either BC related or other causes related. BCSM and OCSM were considered two competing events. The Fine and Gray competing risk analysis (based on the subdistribution hazard ratio [SHR])^11^, ^13^, ^14^ was used to predict the probabilities of the two competing mortality outcomes (BCSM and OCSM). The Fine and Gray model is a multivariable time‐to‐event model, which accounts for the fact that individuals can only have one of the two competing events. The model also accounts for censoring among those who did not have an event during the follow‐up.

The independent risk factors identified in the multivariate analysis were incorporated into the nomogram to predict the probability of 3‐, 4‐, and 5‐year OS, breast cancer‐specific survival (BCSS), and other cause‐specific survival (OCSS) in patients with early‐stage BC using the rms and mstate packages in the R Project.^10^, ^15^ The ability and calibration of the nomogram were assessed by concordance index (C‐index) and calibration curves (comparing the nomogram‐predicted probability with the observed probability).^16^, ^17^ The calibration curves were used to reduce the overfit bias via a bootstrap method with 1000 resamples.^18^ Furthermore, the precision of the 3‐, 4‐, and 5‐year OS, BCSM, and OCSM was evaluated and compared using the area under the receiver operating characteristic curve (AUC). Higher C‐index and AUC values show higher ability to distinguish patients from different survival outcomes. Finally, Kaplan‐Meier curves were plotted for patients grouped by risks predicted from the nomogram to further assess calibration.^18^ A two‐tailed *P*‐value < .05 was considered statistically significant. All analyses were conducted using the R software (version 3.4.3; R Foundation).

## 3 RESULTS

### 3.1 Patient

A total of 196 304 patients with early‐stage BC from 2010 to 2016 were included in the final analysis and randomly divided into two groups at a ratio of 9:1: training cohort (N = 176 674) and validation cohort (N = 19 630). The baseline characteristics of the two groups are presented in Table 1, and there was no significant difference between them (*P* > .05). The median age at diagnosis was 60 years (IQR, 51‐70 years). In the entire population, nearly half of the patients (47.5%) were aged <60 years. Moderate differentiation (Grade II) (44.4%) accounted for the highest proportion, followed by poor differentiation (Grade III‐IV) (30.3%), and good differentiation (Grade I) (25.3%). Small tumors prevailed in patients with early‐stage BC. Regarding size, 62.7% of the tumors were smaller than 2 cm, and only 2.8% were larger than 5 cm. Most patients (75.1%) were categorized as having luminal A subtype (hormone receptor [HR]+/human epidermal growth factor receptor‐2 [HER2]−), followed by triple negative subtype (HR−/HER2−) (10.8%), luminal B subtype (HR+/HER2+) (10.2%), and HER2‐enriched subtype (HR−/HER2+) (3.9%).

### 3.2 Survival

The median follow‐up duration was 41 months (IQR, 24‐60 months). Of 196 304 patients, 12 417 (6.3%) died: 5628 (45.3%) due to BC and 6789 (54.7%) due to other causes. The 3‐, 4‐, and 5‐year OS, BCSM, and OCSM rates, which were stratified by age, grade, tumor size, subtype, and surgery, are shown in Table 2.

**TABLE 2:** Overall survival rates and cumulative incidences of mortality among patients with breast cancer

	Overall survival rate (%)			Breast cancer‒specific mortality (%)			Other causes‐specific mortality (%)		
	3‐y	4‐y	5‐y	3‐y	4‐y	5‐y	3‐y	4‐y	5‐y
Age (y)									
<60	0.974	0.962	0.952	0.020	0.029	0.038	0.006	0.008	0.010
60‐65	0.970	0.958	0.946	0.016	0.024	0.030	0.014	0.019	0.024
66‐70	0.960	0.944	0.927	0.018	0.025	0.031	0.021	0.031	0.041
71‐75	0.946	0.923	0.898	0.020	0.028	0.038	0.034	0.048	0.065
76‐80	0.909	0.870	0.827	0.032	0.042	0.055	0.059	0.088	0.119
81‐85	0.839	0.777	0.708	0.049	0.064	0.073	0.112	0.159	0.219
>85	0.683	0.582	0.474	0.091	0.116	0.135	0.226	0.302	0.391
Grade									
I	0.969	0.955	0.938	0.005	0.008	0.011	0.025	0.037	0.051
II	0.958	0.939	0.920	0.014	0.021	0.028	0.028	0.039	0.052
III‐IV	0.923	0.897	0.871	0.052	0.069	0.085	0.025	0.034	0.043
Tumor size (cm)									
<1	0.978	0.966	0.954	0.006	0.008	0.012	0.016	0.025	0.035
1‐1.9	0.964	0.948	0.930	0.013	0.019	0.024	0.024	0.034	0.046
2‐2.9	0.939	0.913	0.888	0.030	0.044	0.056	0.031	0.043	0.056
≥3	—	—	—	—	—	—	0.042	0.055	0.067
3‐3.9	0.903	0.868	0.837	0.057	0.079	0.099	—	—	—
≥4	0.876	0.840	0.806	—	—	—	—	—	—
4‐4.9	—	—	—	0.076	0.100	0.120	—	—	—
≥5	—	—	—	0.086	0.105	0.126	—	—	—
Subtype									
HR−/HER2− (triple negative)	0.892	0.860	0.834	0.080	0.102	0.120	0.028	0.038	0.046
HR−/HER2+ (HER2 enriched)	0.936	0.915	0.895	0.041	0.056	0.068	0.022	0.029	0.037
HR+/HER2− (luminal A)	0.958	0.940	0.920	0.014	0.022	0.029	0.027	0.038	0.051
HR+/HER2+ (luminal B)	0.957	0.938	0.920	0.021	0.031	0.040	0.022	0.031	0.040
Surgery to primary sites									
No	0.731	0.664	0.617	0.146	0.184	0.211	0.123	0.152	0.172
Yes	0.956	0.937	0.917	0.020	0.029	0.037	0.024	0.034	0.046

Abbreviations: HER2, human epidermal growth factor receptor type 2; HR, hormone receptor.

**FIGURE 2:**
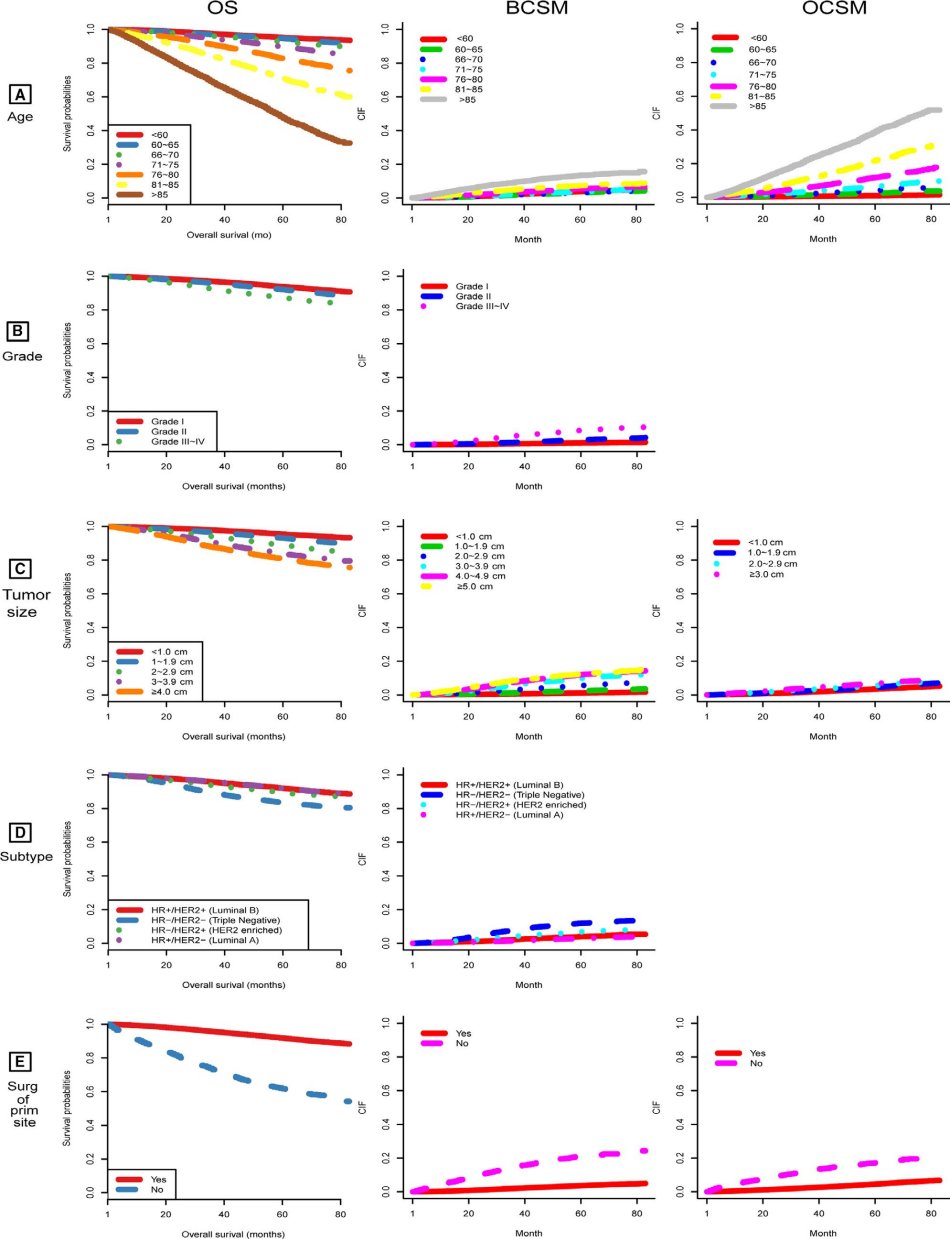
OS and CIF for breast cancer‒specific death and other causes‐specific death according to patient characteristics (A) age; (B) grade; (C) tumor size; (D) subtype; (E) surgery. BCSM, breast cancer‐specific mortality; CIF, cumulative incidence function; HER2−, human epidermal growth factor receptor type 2 negative; HER2+, human epidermal growth factor receptor type 2 positive; HR−, hormone receptor negative; HR+, hormone receptor positive; OCSM, other‐causes‐specific mortality; OS, overall survival; Surg of prim site, surgery of primary site

In the multivariable Cox regression analysis (Table 3), older age (*P* < .001), poorer differentiation (Grade II vs Grade I, hazard ratio [HR], 1.135; 95% confidence interval [CI], 1.075‐1.199; *P* < .001; Grade III–IV vs Grade I, HR, 1.703; 95% CI, 1.602‐1.809; *P* < .001), larger tumor size (*P* < .001), triple negative subtype (vs luminal B subtype, HR, 1.859; 95% CI, 1.724‐2.004; *P* < .001), HER2‐enriched subtype (vs luminal B subtype, HR, 1.167; 95% CI, 1.048‐1.300; *P* = .005), and absence of surgery (vs surgery, HR, 3.428; 95% CI, 3.277‐3.641; *P* < .001) were significantly associated with poorer OS. For BCSM and OCSM, the Fine and Gray competing risk analysis was used, and the following factors were validated (Table 3): older age, poorer differentiation, larger tumor size, triple negative subtype, HER2‐enriched subtype, and absence of surgery for BCSM and older age, larger tumor size, and absence of surgery for OCSM. Age was a strong predictive factor and more obvious in OCSM (*P* < .001). The OCSM rate was significantly increased in patients with increasing age compared with young patients. Elderly patients had higher competing risks (60‐65 years vs <60 years, SHR, 2.561, 95% CI, 2.283‐2.873; *P* < .001; 66‐70 years vs <60 years, SHR, 4.182, 95% CI, 3.742‐4.674; *P* < .001; 71‐75 years vs <60 years, SHR, 6.727, 95% CI, 6.049‐7.482; *P* < .001; 76‐80 years vs <60 years, SHR, 12.008, 95% CI, 10.855‐13.284; *P* < .001; 81‐85 years vs <60 years, SHR, 22.167, 95% CI, 20.112‐24.433; *P* < .001; >85 years vs <60 years, SHR, 39.263, 95% CI, 35.634‐43.262; *P* < .001). The impact of surgery was more prominent on BCSM. Increasing tumor size was correlated with OS and BCSM and slightly correlated with OCSM. Notably, grade and subtype differences were more predominant in BCSM than in OCSM. The Kaplan‐Meier curves for OS and cumulative incidence curves for BCSM and OCSM are presented in Figure 2.

**TABLE 3:** Outcomes of multivariate Cox analysis for OS, Fine and Gray's analysis for BCSM and OCSM

	OS			BCSM			OCSM		
	HR	95% CI	*P*‐value	SHR	95% CI	*P*‐value	SHR	95% CI	*P*‐value
Age (y)									
<60	reference	—	—	reference	—	—	reference	—	—
60‐65	1.416	1.322‐1.516	***	1.082	0.989‐1.184	***	2.561	2.283‐2.873	***
66‐70	2.105	1.879‐2.162	***	1.306	1.181‐1.443	***	4.182	3.742‐4.674	***
71‐75	2.900	2.707‐3.107	***	1.545	1.389‐1.717	***	6.727	6.049‐7.482	***
76‐80	4.914	4.604‐5.245	***	2.208	1.988‐2.453	***	12.008	10.855‐13.284	***
81‐85	8.292	7.878‐8.829	***	2.780	2.493‐3.099	***	22.167	20.112‐24.433	***
>85	14.169	13.340‐15.050	***	3.852	3.469‐4.276	***	39.263	35.634‐43.262	***
Grade									
I	reference	—	—	reference	—	—	—	—	—
II	1.135	1.075‐1.199	***	2.084	1.845‐2.353	***	—	—	—
III‐IV	1.703	1.602‐1.809	***	4.486	3.956‐5.087	***	—	—	—
Tumor size (cm)									
<1	reference	—	—	reference	—	—	reference	—	—
1‐1.9	1.445	1.355‐1.541	***	1.717	1.516‐1.944	***	1.343	1.247‐1.447	***
2‐2.9	2.116	1.978‐2.263	***	3.041	2.686‐3.445	***	1.681	1.551‐1.822	***
≥3	—	—	—	—	—	—	1.869	1.718‐2.034	***
3‐3.9	2.820	2.619‐3.037	***	4.589	4.030‐5.226	***	—	—	—
≥4	3.307	3.069‐3.565	***	—	—	—	—	—	—
4‐4.9	—	—	—	5.539	4.810‐6.380	***	—	—	—
≥5	—	—	—	5.851	5.032‐6.804	***	—	—	—
Subtype									
HR+/HER2+ (luminal B)	reference	—	—	reference	—	—	—	—	—
HR−/HER2− (triple negative)	1.859	1.724‐2.004	***	2.460	2.219‐2.727	***	—	—	—
HR−/HER2+ (HER2 enriched)	1.167	1.048‐1.300	**	1.384	1.200‐1.595	***	—	—	—
HR+/HER2− (luminal A)	1.000	0.934‐1.071	0.996	1.065	0.960‐1.181	0.230	—	—	—
Surgery to primary sites									
Yes	reference	—	—	reference	—	—	reference	—	—
No	3.428	3.227‐3.641	***	4.031	3.687‐4.406	***	2.037	1.851‐2.242	***
C‐index (95%CI)	0.801 (0.795, 0.806)			0.830 (0.824, 0.836)			0.806 (0.798, 0.814)		

Abbreviations: BCSM, breast cancer‒specific mortality; CI, confidence interval; HER2−, human epidermal growth factor receptor type 2 negative; HER2+, human epidermal growth factor receptor type 2 positive; HR, hazard ratio; HR−, hormone receptor negative; HR+, hormone receptor positive; OCSM, other causes‐specific mortality; OS, overall survival; SHR, subdistribution hazard ratio.

** *P* < .05.

*** *P* < .001.

### 3.3 Nomogram

**FIGURE 3:**
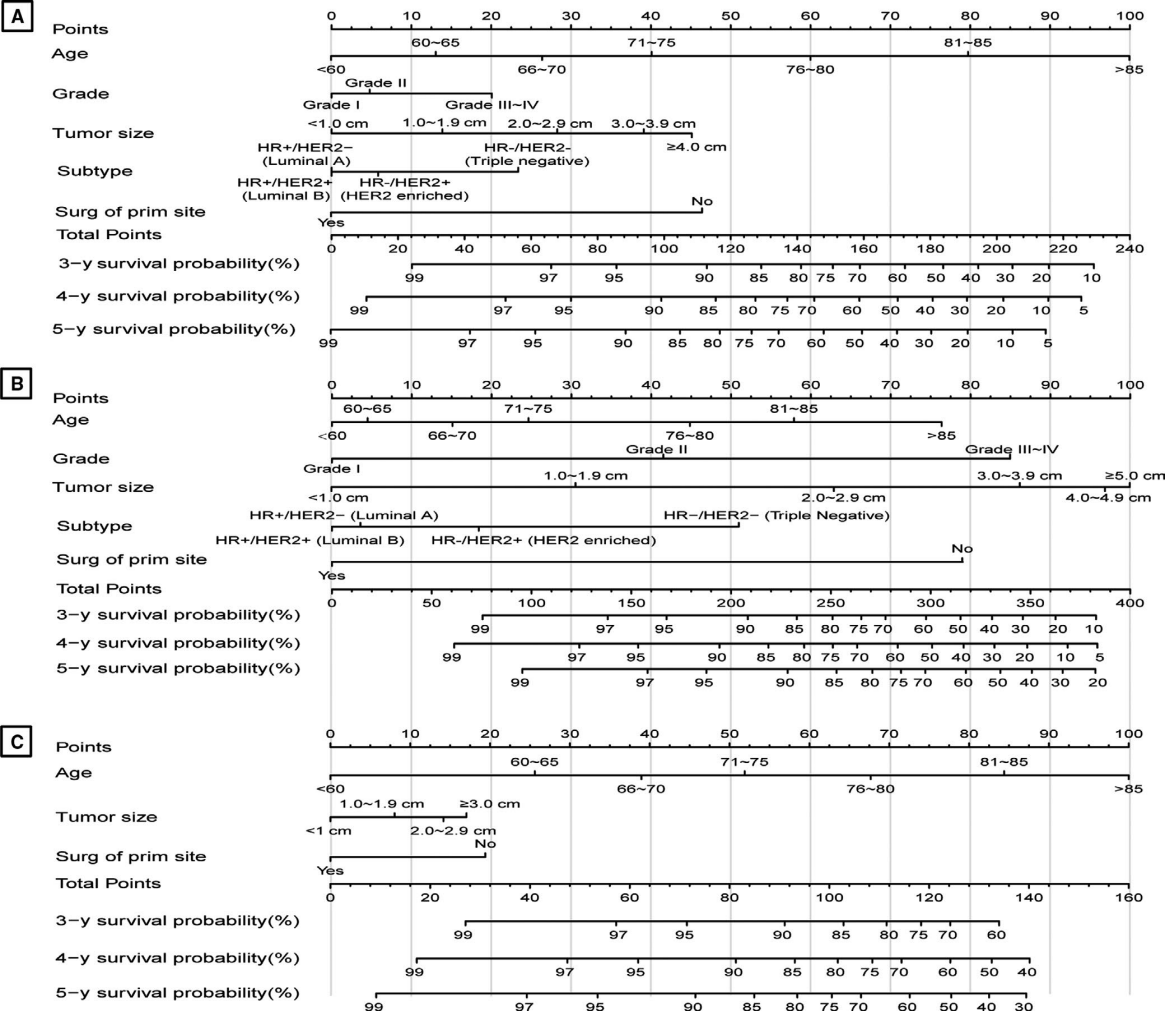
Nomograms predicting 3‐, 4‐, and 5‐y OS (A), BCSS (B), and OCSS (C). BCSS, breast cancer‐specific survival; HR−, hormone receptor negative; HR+, hormone receptor positive; HER2−, human epidermal growth factor receptor type 2 negative; HER2+, human epidermal growth factor receptor type 2 positive; OCSS, other causes‐specific survival; OS, overall survival; Surg of prim site, surgery of primary site

**FIGURE 4:**
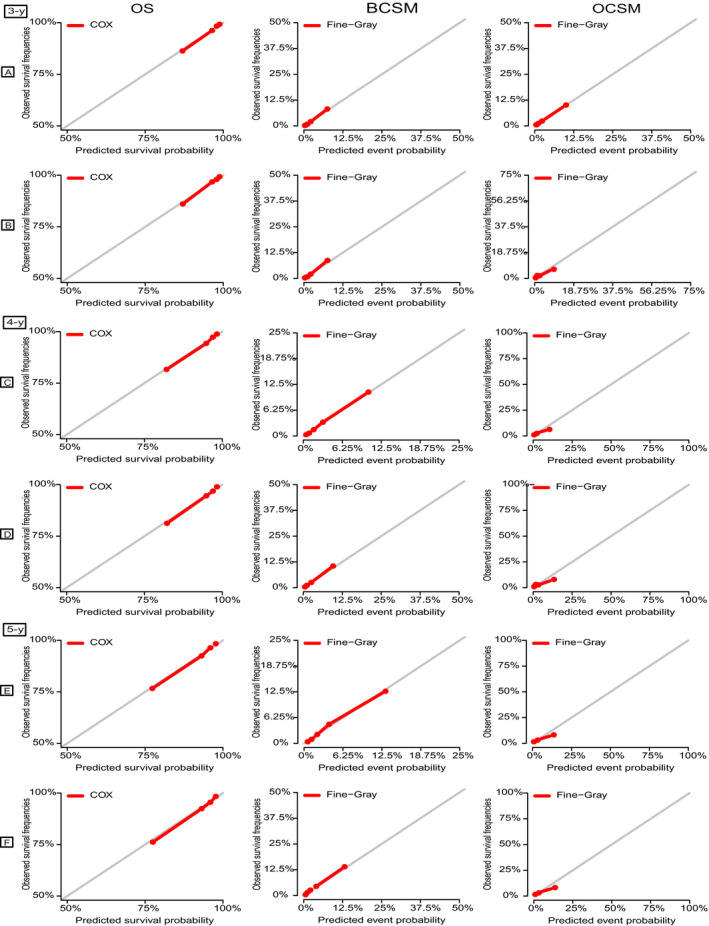
Calibration plots of the nomograms for 3‐, 4‐, and 5‐y OS, BCSM, and OCSM. A, C, E, Calibration plots of the training cohort; (B, D, F) Calibration plots of the validation cohort. *X*‐axis represents the nomogram‐predicted event probabilities; *Y*‐axis represents the observed event frequencies. BCSM, breast cancer‒specific mortality; OCSM, other causes‐specific mortality; OS, overall survival

Five validated variables were incorporated to develop the prognostic nomogram: age, grade, tumor size, subtype, and surgery at the primary site (Figure 3). Thus, the probability of 3‐, 4‐, and 5‐year OS, BCSS, and OCSS could be predicted by summing up the scores of each selected variable (higher total points, worse prognosis), helping to identify patients with high risk of BC or other causes of death. The nomogram demonstrated considerably strong discriminative ability, with a C‐index of 0.801 (95% CI, 0.795‐0.806; *P* = .003) for the OS model (using Cox regression analysis), 0.830 (95% CI, 0.824‐0.836; *P* = .003) for the BCSM model, and 0.806 (95% CI, 0.798‐0.814; *P* = .004) for the OCSM model (using Fine and Gray competing risk analysis). Calibration plots presented high conformance between the nomogram‐predicted and observed probabilities in both the training and validation cohorts (Figure 4).

**FIGURE 5:**
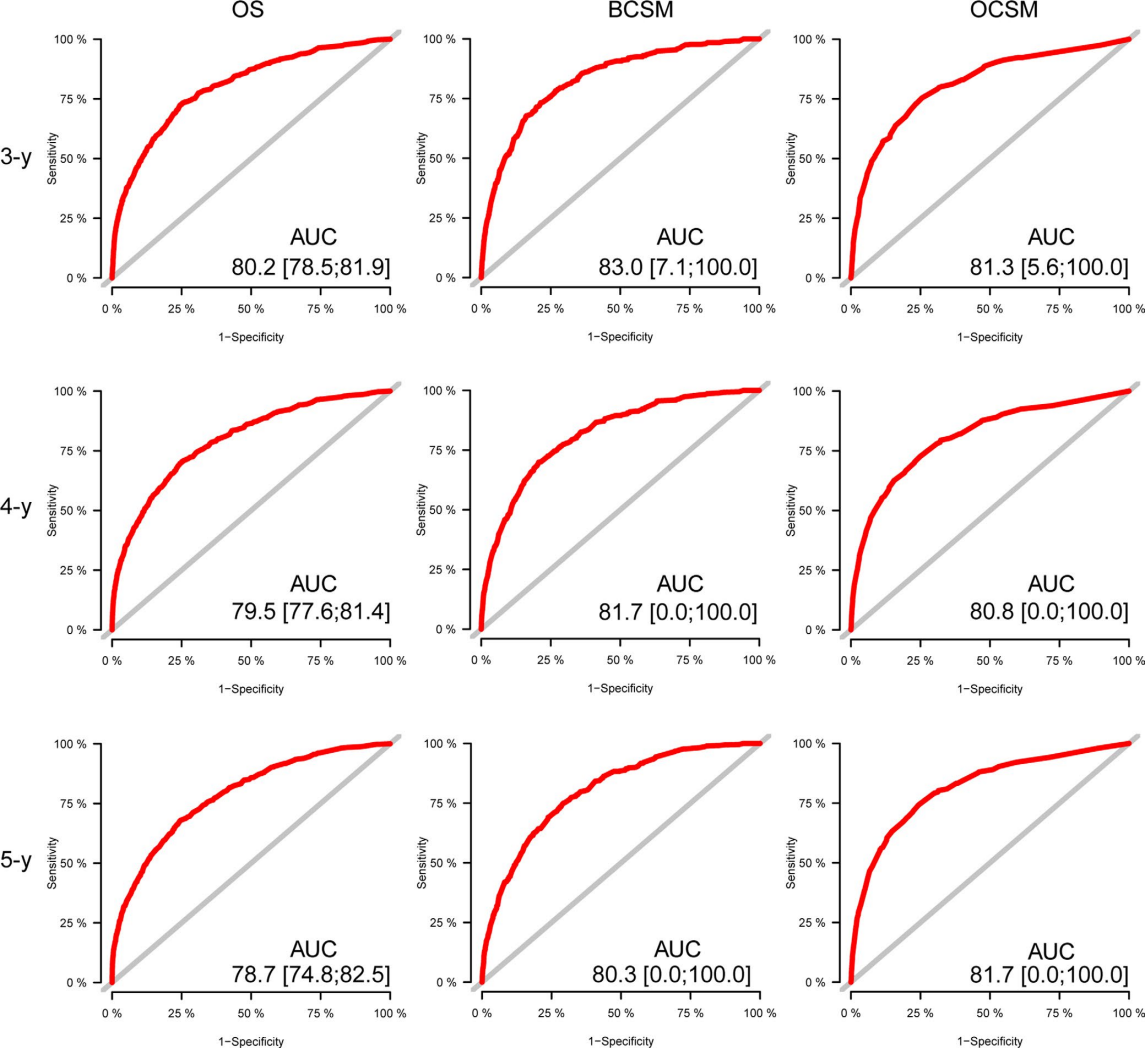
ROC curves for 3‐, 4‐, and 5‐y OS prediction, BCSM prediction, and OCSM prediction. AUC, area under ROC; BCSM, breast cancer‒specific mortality; OCSM, other causes‐specific mortality; OS, overall survival; ROC, receiver operating characteristic curve

The discriminatory capacity of the nomogram was evaluated by calculating the AUC values (Figure 5). The AUC values for predicting 3‐, 4‐, and 5‐year OS were 80.2%, 79.5%, and 78.7%, respectively. As for the prediction of the 3‐, 4‐, and 5‐year BCSM, the AUC values were 83.0%, 81.7%, and 80.3%, respectively. Moreover, the AUC values were 81.3%, 80.8%, and 81.7%, respectively, for the 3‐, 4‐, and 5‐year OCSM.

Based on the C‐index and AUC values, the model predicting BCSM and OCSM using the Fine and Gray competing risk analysis had more precision than that of predicting OS.

**FIGURE 6:**
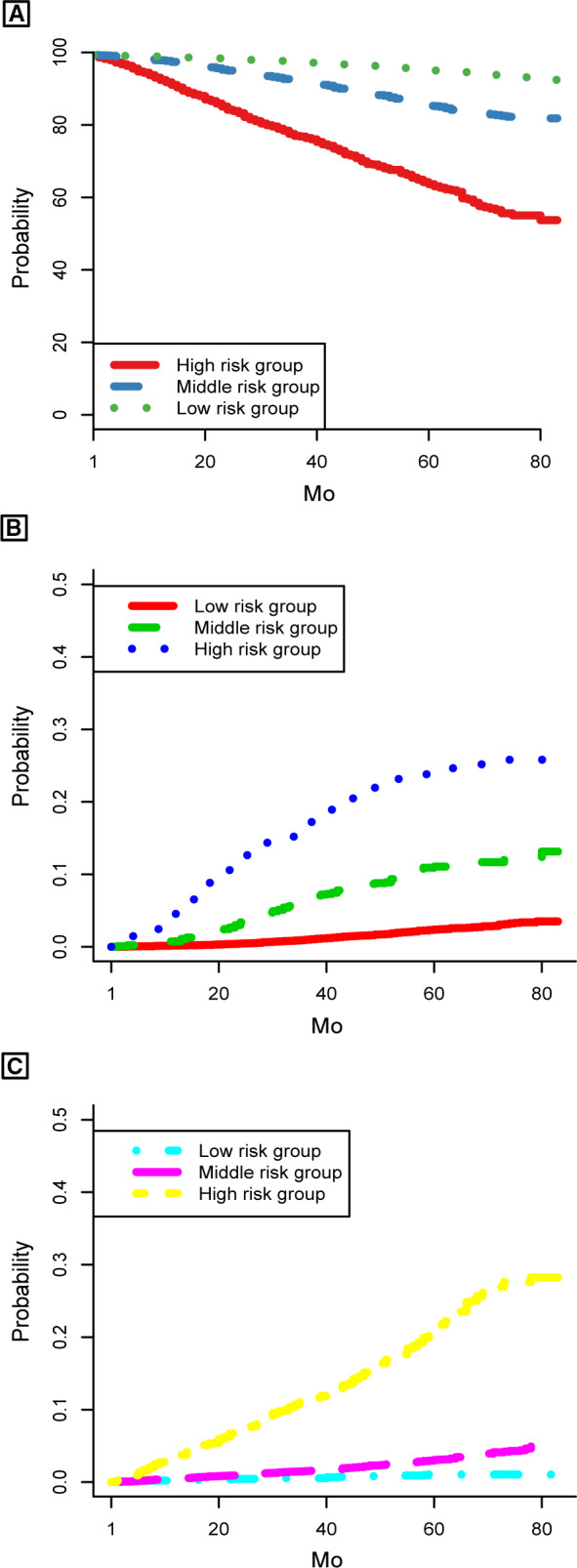
Kaplan‐Meier and CIF curves stratified by the risk levels of nomogram‐predicted probabilities. A, OS rates; (B) BCSM; (C) OCSM. BCSM, breast cancer‒specific mortality; OCSM, other causes‐specific mortality; OS, overall survival

Furthermore, to further evaluate the discrimination of the model, the validation cohort was stratified into three groups based on the predicted probability calculated from the nomogram: low‐, middle‐, and high‐risk groups. Among the entire population, patients in the high‐risk group had significantly lower OS rates and higher BCSM or OCSM rates than patients in the low‐ and middle‐risk groups (5‐year OS rate: 0.644 for high‐risk group, 0.860 for middle‐risk group and 0.958 for low‐risk group; 5‐year BCSM rate: 0.238 for high‐risk group, 0.111 for middle‐risk group and 0.024 for low‐risk group; 5‐year OCSM rate: 0.213 for high‐risk group, 0.031 for middle‐risk group and 0.010 for low‐risk group) (*P* < .001) (Figure 6).

## 4 DISCUSSION

In the study, we analyzed the survival and mortality in patients with early‐stage BC, discriminating the differences between BC‐related and other cause‐related risk factors. A comprehensive nomogram was built to predict OS, BCSS, and OCSS as a convenient clinical tool.

To the best of our knowledge, this study was based on more than 190,000 patients from the SEER database, which contains the largest cohort to date. It is the first study to use the Fine and Gray competing risk analysis based on the proportional SHR to model the CIF.^13^, ^19^ Unlike previous nomograms,^20^, ^21^ providing the physician with a patient's probability of surviving the disease assuming no death from a competing cause, our nomogram is comprehensive, considering OCSM, and shows relatively good calibration and discrimination power with C‐indices > 0.80 and AUC values of approximately 80%.

From 2010 to 2016, 12 417 (6.3%) of 196 304 patients died, of whom 5628 (45.3%) had BCSM and 6789 (54.7%) had OCSM.

Although the follow‐up duration was insufficient, more than half of the deaths were attributed to causes other than primary BC. It is better to consider such competing risks when evaluating prognosis for decision‐making and patient counseling.

Age was a strong predictive factor and more obvious in OCSM. That is, older patients had higher risk of OCSM. Chen et al^9^ also revealed that elderly women exhibited worse OS but better BCSS than young women, although OCSM was not evaluated. These results may be due to higher frequencies of age‐related comorbidities and less basic life support, leading to high OCSM. Therefore, in patients with early‐stage BC, it is equally important to pay attention to the primary breast and age‐related diseases. A healthy lifestyle that includes weight management, self‐care, and preventive strategies should be encouraged by physicians to prevent OCSM.

The far‐reaching impact of surgery was observed, especially on BCSM. In our study, based on those who underwent surgery, regardless of the surgery type, patients who did not undergo surgery had significantly poorer prognosis. Almost 90% of women diagnosed with BC have early‐stage disease and may be treated with breast‐conserving surgery or mastectomy.^22^, ^23^ The long‐term survival of women with early BC who were treated with breast‐conserving surgery and postoperative radiotherapy was virtually identical to that in women who underwent radical mastectomy.^24^ However, surgery itself may carry a series of risks and adverse effects, leading to the increase in OCSM rate.

Although this study presents a good predictive nomogram, there are still several limitations. First, due to the unavailable subtype information before 2010 in the SEER database, the follow‐up (2010‐2016) duration was short for early‐stage BC. A longer follow‐up duration may improve the precision and discrimination of our model. Second, the variable of comorbidity is lacking. SEER does not collect data on comorbid status, which worsens with age and affects patient survival. Instead, we consider age as a replacement of the comorbidity to compensate for the limitation. Finally, internal validation was used to evaluate the model. Although it demonstrated good accuracy, external validation based on other patient cohorts is still needed.

## 5 CONCLUSIONS

We evaluated OS and competing risks of death in patients with early‐stage BC based on the Fine and Gray competing risk analysis. This is the first study to develop a comprehensive nomogram predicting 3‐, 4‐, and 5‐year OS, BCSS, and OCSS using a large population. Additionally, the well‐performed nomogram may help answer patients’ consultation questions and offer prognostic assessment for individuals. However, more studies are required for further external validation.

## CONFLICT OF INTEREST

The authors have declared that no competing interest exists.

## AUTHOR CONTRIBUTIONS

Yan‐Bo Xu and Dong Xu designed the study. Yan‐Bo Xu, Hong Liu, and Qi‐Hua Cao performed literature review, data collection, and analyzed the data. Yan‐Bo Xu and Hong Liu contributed to manuscript drafting. Dong Xu, Jia‐Li Ji, and Rong‐Rong Dong critically revised the manuscript.

## ETHICAL STATEMENT

Not applicable.

## Data Availability

The data that support the findings of this study are openly available in the Surveillance, Epidemiology, and End Results, https://seer.cancer.gov.
